# The prevalence of Y chromosome microdeletions in Pakistani infertile men

**Published:** 2013-08

**Authors:** Rubina Tabassum Siddiqui, Nosheen Mujtaba, Mamoona Naz

**Affiliations:** 1 *Health Biotechnology Division, National Institute for Biotechnology and Genetic Engineering (NIBGE), Faisalabad, Pakistan.*; 2 *Institute of Biochemistry and Biotechnology, University of the Punjab, Quaid-e-Azam Campus, Lahore, Pakistan.*

**Keywords:** *Male infertility*, *Azoospermia*, *Y Chromosome deletions*

## Abstract

**Background:** Microdeletions of the azoospermia factor locus of the long arm of Y chromosome are an etiological factor of severe oligozoospermia or azoospermia.

**Objective:** The aim of this study was to investigate the prevalence of Y-chromosome microdeletions in AZF region and their role in infertility in Pakistani population.

**Materials and Methods:** The type of deletions in AZF locus were detected in infertile men (n=113) and the association of Y chromosome microdeletions with male infertility was assessed by including men (50) with normal karyotype and having children. Y chromosome microdeletions were detected by multiplex PCR using 10 sequence tagged sites namely sY81, sY130, sY141, sY142, sY155, sY157, sY160, sY182, sY231, and sY202 that covered all three regions of AZF.

**Results:** Individuals with severe oligozoospermia showed 2.86% deletion frequency in AZFc region as compared to azoospermic males (5.5%).

**Conclusion:** The results of our study showed that deletions in Y chromosome are not playing major part in male infertility. Moreover, multiplex-PCR strategy might preferably be employed for the detection of Y chromosome microdeletions allied to male infertility.

## Introduction

Although the human desire to have children is deeply ingrained, as many as 15% of all couples experience some form of infertility (1, 2). After the Klinefelter syndrome, Y chromosome microdeletions are the most frequent genetic cause of male infertility (3). However, the origin of reduced male fertility is still unknown in approximately 30% of cases. Azoospermia and severe oligozoospermia can be associated with many conditions such as sperm duct obstruction, cryptorchidism, endocrine disorders, infection, chromosome abnormalities, and submicroscopic deletions in the long arm of Y chromosome (4, 5). 

There is no good animal model to study the genes related to male infertility. Therefore the presence of Y chromosome microdeletions may be considered as naturally occurring ‘knockouts’ for deleted genes. Deletions in Y chromosome are associated with deletions of AZF region genes. This causes multiple downstream effects on expression of a large number of genes in testes. Hence, AZF transcriptome and consequently the proteins encoded by this region affect the spermatogenesis. AZFa region is located in proximal Yq and is between 1 and 3 Mb in size and contains four single copy genes namely *USP9Y, DBY, UTY* and *TB4Y*. 

The* DBY* gene is involved in translation of mRNA in testes and particularly in spermatogonia and pachytene spermatocytes. The AZFb region is 1.3 Mb and contains eight protein coding genes (*CDY2, EIFIAY, HSFY, PRY, RBMYLI, RPS4YS, SMCY,* and* XKRY*). These are all transcribed in testicular tissue and perform their role in spermatogenesis. The *AZFc* contains five genes (*BPY2, CDYI, CSPLY, DAZ *and* GOLGA2LY*) (6-8). The “Deleted in Azoospermia” (*DAZ*) gene family is reported to be the most frequently deleted *AZF* candidate genes and is located in the *AZFc* region (9). The *DAZ* gene family encodes proteins that are expressed in testicular tissues. These proteins contain an RNA recognition motif and have regulatory role in RNA metabolism (10). 

Men with *DAZ* deletions either produce no sperms or may have oligospermia (11). With the advent of assisted reproductive technology, it has been made possible for men with a cytogenetic abnormality or microdeletions on the Y chromosome to father children with intracytoplasmic sperm injection (ICSI) treatment. However, there is a chance that the abnormality will be transmitted to the male offspring of these men, causing infertility in future generations (12). 

The present study was designed to establish an STSs based multiplex polymerase chain reaction (PCR) assay for amplification of AZFa, AZFb, AZFc, and *DAZ* gene simultaneously to investigate the type of Y chromosome microdeletions and their prevalence in infertile Pakistani men. The data obtained will be helpful in understanding the possible causes of male infertility in Pakistan and in better management of these cases. The better the understanding of molecular mechanisms regulating spermatogenesis, the faster and more accurate will be the diagnosis.

## Materials and methods

**Subjects**

A cross sectional study was planned to work out the prevalence of y chromosome microdeletions in infertile men. For that, men with fertility problems from different hospitals and infertility clinics in three most populated provinces (Punjab, Sindh and Khyber Pakhtunkhwa) of Pakistan were included in the study. Individuals with spermatogenic failure were included in the study. Patients selection criteria of azoospermia and oligozoospermia was made on the basis of semen analysis, according to World Health Organization standard as <20×10^6^ sperm/ml (13). Those with obstructive azoospermia and abnormal karyotype were excluded from the study. 

Finally, 113 infertile men were screened for the presence or absence of Y chromosome microdeletions. A control group comprising of 50 healthy, normozoospermic, fertile men having two or more children was also included in the study. Informed consent was obtained from each subject prior to collecting samples. The infertile men ranged from 24-50 years in age (mean=34). Both patients and controls were of similar age group and were all native Pakistanis. This study was approved by the ethical committee of the National Institute of Biotechnology and Genetic Engineering, Faisalabad, Pakistan.

**Cytogenetic evaluation**

Peripheral blood lymphocytes of each individual were cultured in complete RPMI 1640 medium (10% fetal calf serum) at 37^o^C for 72 hours. The lymphocytes were proliferated with 2% phytohaemaglutinin. Chromosomes were arrested at metaphase with 0.1 μg/mL of colcemid (Seromed, Berlin, Germany) after 71-h incubation period before harvesting as described earlier (14). Cells were given hypotonic treatment with 0.075M KCl for 6 minutes at 37^o^C. 

The cells were then fixed with methanol: glacial acetic acid (3:1) for 10 minutes. Slides were prepared and stained using standard G-banding technique. At least 30 metaphases per subject were analyzed using an automated system of chromosome analysis (Genetic) CV Chromoscan. Only patients with normal karyotype (46, XY) as shown by Giemsa-trypsin-Giemsa (GTG) banding were included in this study.

**DNA extraction from peripheral blood**

Peripheral blood samples (3 ml) were collected in EDTA vacutainer tubes and genomic DNA was extracted using phenol-chloroform method (15). Blood samples (750 µl) were taken in microfuge tube and mixed with 750 µl of solution A (0.32 M sucrose, 10 mM Tris-HCl pH 7.5, 5 mM MgCl_2_ and 1% v/v Triton X100) for 5 minutes at room temperature. The mixture was then centrifuged at 13,000xg for 5 minutes. 

The pellet was then resuspended in 400 µl of solution B (10 mM Tris-HCl pH 7.5, 400m M NaCl, 2 mM EDTA, pH 8.0). The cell suspension was treated with 12 µl of 20% SDS and 5 µl of proteinase-K (15 mg/ml) solution and incubated at 37^o^C overnight or at 65^o^C for one hour. The DNA was then extracted with phenol and chloroform-isoamyl alcohol. 

DNA was precipitated by adding 1/10 volume of sodium chloride (3 M, pH 6.0) and 1.5 volume of chilled isopropanol. DNA pellet was washed with 70% ethanol, air-dried and resuspended in 150 µl of Tris-EDTA (pH 8.0). Qualitative analysis of DNA was carried out by 0.8% agarose gel electrophoresis and quantification of DNA was done by spectrophotometrically (NanoDrop), Bio Rad.

**PCR amplification of STS**

The *AZF* candidate genes on chromosome Y of azoospermic, severe oligospermic, moderate oligospermic and control men, were amplified by PCR using primers specific for STSs in Y chromosome as mentioned in table I (16). Two primer pairs sY81, sY160 were used to amplify AZFa region, Two primer pairs sY130, sY182 were used to amplify AZFb region, four primers pairs sY157, sY141, sY155, sY142 were used to amplify in AZFc region in two multiplex reactions. Two primers sY231 and sy202 were used for *DAZ* gene amplification in AZFc region, sY4 was used as internal control. PCR was carried out in 25 µl reaction volume containing; 1X PCR buffer; 4.5 mM MgCl_2_; 0.4 mM dNTPs mix; 0.5 µM of each primer, 1U Taq DNA polymerase and 200 ng of genomic DNA. 

Amplification was carried out for 35 cycles in a Perkin Elmer thermal cycler as follows: denaturation at 94^o^C for 1 min, annealing at 61^o^C for 1min, and extension at 72^o^C for 1min. Amplification cycles were proceeded by initial denaturation for 5 min at 95^o^C and final extension at 72^o^C for 5 min. All reagents were purchased from Fermentas (USA) and primers were made from Operon, Germany. Amplification products were resolved on 3% agarose gel. In each PCR reaction, DNA from a normal fertile male was used as normal control, while female DNA and water served as negative control. 

A 50bp DNA marker (Fermentas) was loaded with PCR products to confirm the band size. Gel was stained with ethidium bromide, visualized under UV (UVitec Gel Documentation System) and photographed. A positive result was scored when the amplification product of expected size was obtained. Deletion of one or more PCR fragments was confirmed by amplification using a single pair of primers and was repeated thrice. 

## Results

**Figure 1:**
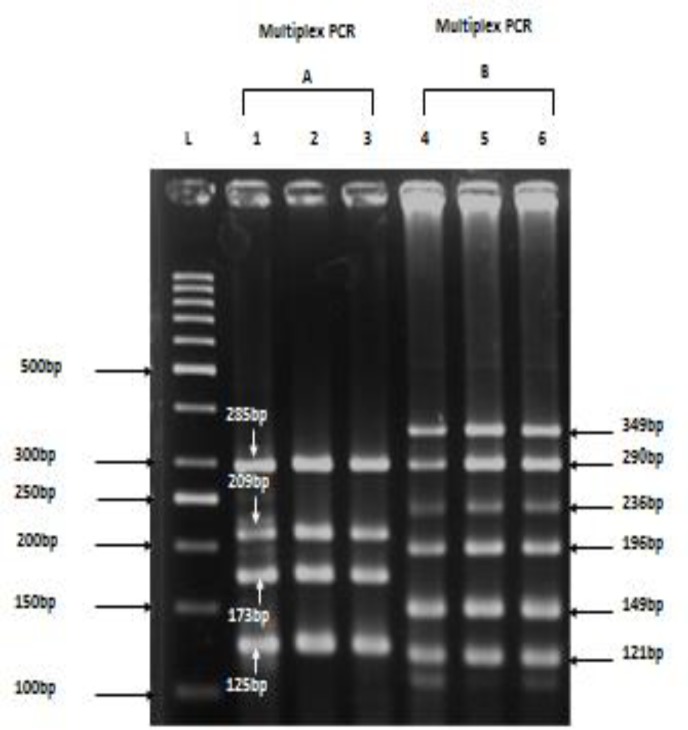
Resolution of multiplex PCR products of ten STS of Y-chromosome DNA on 3 % agarose gel. Lane L: 50 bp DNA ladder (Gene Ruler; Fermentas #SM0373), Lanes 1-3: amplification products from reaction mixture using Multiplex A master mix: sY81 (209 bp), sY130 (173 bp), sY157(285 bp) and sY182(125 bp), sY231(149bp) and sY202 (121 bp); Lanes 4-6: amplification products from reaction mixture using Multiplex B master mix: sY141(290 bp), sY142 (196 bp), sY155 (349 bp), sY160 (236 bp). Lane1 and 4 are normal fertile individuals and 2,3,4,5 infertile patients

One-hundred sixty three males were analyzed; 50 fertile males with normal semen parameters (mean age 25±1 years) and 113 infertile males (mean age 34±5 years). Infertile males were classified according to concentration of spermatozoa in semen analyses. Twenty four patients had moderate oligospermia, 35 patients showed severe oligospermia, and 54 were azoospermic males (Table II). Chromosome analysis was performed analyzing 30 metaphases for each patient and control. No chromosome abnormality was found in autosomes. Only one sex linked chromosomal aberration (47, XXY) was detected in an oligospermic infertile person. This individual was excluded from the study. Genomic DNA from patients and controls were examined using two sets of multiplex PCR primers (Figure 1) and repeated amplification failure of any particular primer pair suggested a deletion.

**Figure 2:**
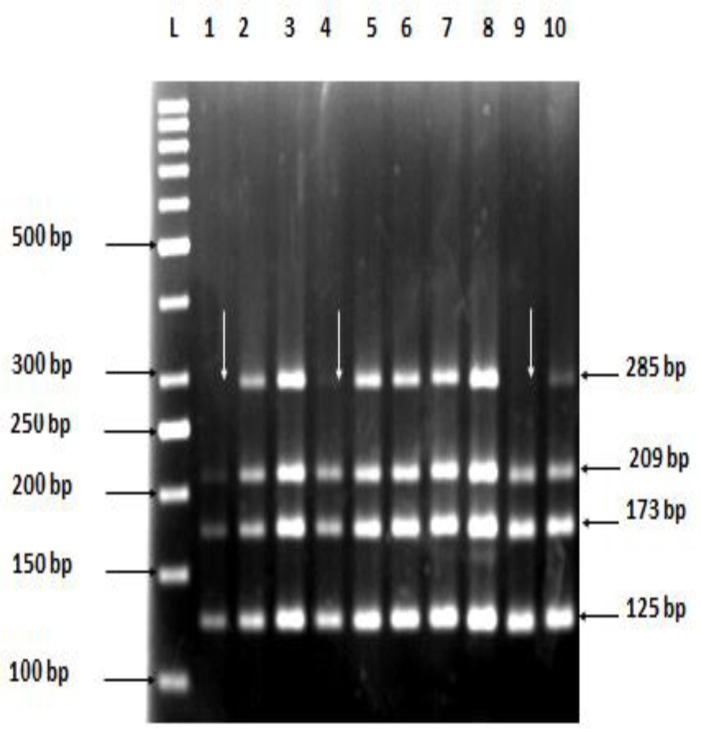
Y-chromosome microdeletions; amplification products from “Multiplex A” master mix resolved on 3% agarose gel. Lane L: 50 bp DNA ladder (Gene Ruler; Fermentas #SM0373), Lane 1-9: Amplification products from DNA of infertile males. Lane 1, 4 and 9 show the absence of sY157 (285bp) *AZFc* deletion, Lane 10: positive control (fertile male).

The results were confirmed as deletions only when both multiplex and single PCR with specific primers showed same results. Submicroscopic deletions of the Y chromosome were detected and confirmed in four patients (4/113) 3.54% with male infertility (Figure 2). The frequency of microdeletions in the azoospermic group was 5.5% (3/54) and in severe oligozoospermic group was 2.86% (1/35). All affected patients had Y microdeletions spanning the proximal *AZFc *sub region adjacent to the *DAZ *gene. These deletions affected contiguous segments of the Y chromosome. None of the individuals from control group showed any microdeletion in Y-chromosome. Hence, PCR multiplex assays are the method of choice for detecting microdeletions in the long arm of the human Y chromosome.

**Table I:** Primer sequence, loci, STS and the expected size of the PCR product

**Multiplex name and Loci**		**STS**	**Forward primer (5'→3'** **)**	**Reverse primer(5'→3'** **)**	**PCR product Size (bp)**
**Multiplex Reaction Mix “A”**					
	DYS240	SY157	CTTAGGAAAAAGTGAAGCCG	CCTGCTGTCAGCAAGATACA	285	
	DYS271	SY81	AGGCACTGGTCAGAATGAAG	AATGGAAAATACAGCTCCCC	209	
	DYS221	SY130	AGAGAGTTTTCTAACAGGGCG	TGGGAATCACTTTTGCAACT	173	
	KAL182	SY182	TCAGAAGTGAAACCCTGTATG	GCATGTGACTCAAAGTATAAGC	125	
**Multiplex Reaction Mix “B”**					
	DYF53S1	SY155	ATTTTGCCTTGCATTGCTAG	TTTTTAAGCCTGTGACCTGG	349
	DYS229	SY141	GCAGTTCCATTGTTTGCTTC	GCAGCATAATAGCTATACAGTATGG	290
	DYZ1	SY160	TACGGGTCTCGAATGGAATA	TCATTGCATTCCTTTCCATT	236
	DYS230	Y142	AGCTTCTATTCGAGGGCTTC	CTCTCTGCAATCCCTGACAT	196
	DAZ (3)	SY231	ATTGATGTGTTGCCCCAAAT	AGAGTGAACTTTAAATCCCAGCC	149
	DAZ (4)	SY202	ACAGTTTGAAATGAAATTTTAAATGTGTT	TGACAAAGTGAGACCCTACTACTA	121

**Table II:** Frequency of Yq deletions in infertile men

**Classification**	**No. of infertile male individuals (n=103)**	**Sperm concentration** **(/ml)**	**Deletion frequency** **(%)**
Azoospermia	54	0-1×10^6^	5.55
Severe oligozoospermia	35	>1-5×10^6^	2.86
Moderate oligozoospermia	24	>5-20×10^6^	0

## Discussion

Spermatogenesis is a complex process and it is subject to the influence of many genes. In the present study one chromosomal abnormality was observed with 47XXY. G banded metaphase analyses of all 163 infertile and control cases revealed 46, XY normal karyotype. Previous studies for different populations have shown that the incidence of chromosomal abnormalities in infertile males was between 2.2 and 19.6%. For example one study reported 18.3% Klinefelter syndrome among Kuwaiti men (17-19). 

Microdeletions of the Y chromosome represent an important cause of male infertility and one of the most frequent genetic etiologies of severe testiculopathy. The development of Y-specific STS-based mapping strategy has permitted the rapid screening of a large number of infertile patients for Y chromosome microdeletions. The incidence of Y chromosome microdeletions in different ethnic populations reveals different results. It was reported to be 7% in a study by Martinez *et al*, and 3.3% among Turkish male patients (20, 21). 

Similarly, Alkhalaf also observed the frequency of 7.4% in Kuwaiti population (19). The frequencies of these deletions reported in different studies are not consistent with each other and the relationship between sub-deletions and spermatogenesis is still controversial (22-25). The geographical and ethnic differences might affect these deletions in the *AZF* regions, possibly in both the deletion patterns and the phenotypic expression. The changes in the prevalence are due to either small size or endocrine disorders or an unknown environmental factor may be important in influencing the frequency. 

In the present study the deletions were found only in AZFc region and therefore are in agreement with the literature, where the most commonly affected region reported is *AZFc* (12). This region is located at the distal part of deletion interval 6 (subintervals 6C-6E) on the Y chromosome. The phenotype of the individuals showing microdeletions was heterogeneous because three patients were azoospermic and one was severe oligozoospermic. These results are similar to those where a variable clinical and histological phenotype has been found (26).

The relatively low prevalence of genetic abnormalities (2.86-5.5%) in our study strongly suggests that there might be factors, other than Y chromosome microdeletions, responsible for male infertility. Such genetic alterations would remain undetectable by Y microdeletion testing. The difference in frequency of deletions could also be due to the selection of patients, as we collected samples from infertile men attending all types of clinics and not the ones seeking ICSI. So, our study population might not have severely affected phenotypically than men reported from other countries.

It has been observed that the microdeletions in Yq can lead to progressive worsening of sperm production and that with time oligozoospermic men can become azoospermic (27, 28). Consequently, it is recommended that men with microdeletions acquired by natural transmission or by ICSI be submitted to andrologic examinations in puberty and have their sperm cryopreserved before possible decline in number with age (29). The genetic consequences of the transmission of microdeletions necessitate a precise evaluation of the function of various genes on the Y chromosome, especially those that involve the *AZF* region and that are responsible for male infertility.

## Conclusion

Our results show that the prevalence of Y chromosome microdeletions is not too significant in our population. There might be other factors contributing to male infertility. Multiplex-PCR strategy may be used for the detection of Y chromosome microdeletions in infertile men who want to have children born via ICSI.
